# Proposed minimal diagnostic criteria for myelodysplastic syndromes (MDS) and potential pre-MDS conditions

**DOI:** 10.18632/oncotarget.19008

**Published:** 2017-07-05

**Authors:** Peter Valent, Attilio Orazi, David P. Steensma, Benjamin L. Ebert, Detlef Haase, Luca Malcovati, Arjan A. van de Loosdrecht, Torsten Haferlach, Theresia M. Westers, Denise A. Wells, Aristoteles Giagounidis, Michael Loken, Alberto Orfao, Michael Lübbert, Arnold Ganser, Wolf-Karsten Hofmann, Kiyoyuki Ogata, Julie Schanz, Marie C. Béné, Gregor Hoermann, Wolfgang R. Sperr, Karl Sotlar, Peter Bettelheim, Reinhard Stauder, Michael Pfeilstöcker, Hans-Peter Horny, Ulrich Germing, Peter Greenberg, John M. Bennett

**Affiliations:** ^1^ Department of Internal Medicine I, Division of Hematology & Hemostaseology, Medical University of Vienna, Vienna, Austria; ^2^ Ludwig Boltzmann Cluster Oncology, Medical University of Vienna, Vienna, Austria; ^3^ Department of Pathology and Laboratory Medicine, Weill Cornell Medical College, New York, NY, USA; ^4^ Division of Hematological Malignancies, Department of Medical Oncology, Dana-Farber Cancer Institute, Harvard Medical School, Boston, MA, USA; ^5^ Division of Hematology, Department of Medicine, Brigham and Women’s Hospital, Harvard Medical School, Boston, MA, USA; ^6^ Clinic of Hematology and Medical Oncology, Universitymedicine Göttingen, Göttingen, Germany; ^7^ Department of Molecular Medicine, University of Pavia, Pavia, Italy; ^8^ Department of Hematology Cancer Center Amsterdam, VU University Medical Center, Amsterdam, The Netherlands; ^9^ Munich Leukemia Laboratory (MLL), Munich, Germany; ^10^ Hematologics, Inc., Seattle, WA, USA; ^11^ Department of Internal Medicine II, Marien Hospital, Düsseldorf, Germany; ^12^ Servicio Central de Citometría, Centro de Investigación del Cáncer (IBMCC, CSIC-USAL) and IBSAL, Universidad de Salamanca, Salamanca, Spain; ^13^ Department of Medicine I, Medical Center-University of Freiburg, Freiburg, Germany; ^14^ Department of Hematology, Hemostasis, Oncology, and Stem Cell Transplantation, Hannover Medical School, Hannover, Germany; ^15^ Department of Hematology and Oncology, University Hospital Mannheim, Medical Faculty Mannheim of the University of Heidelberg, Mannheim, Germany; ^16^ Metropolitan Research and Treatment Center for Blood Disorders (MRTC Japan), Tokyo, Japan; ^17^ Laboratoire d’Hématologie CHU de Nantes, Nantes, France; ^18^ Department of Laboratory Medicine, Medical University of Vienna, Vienna, Austria; ^19^ Institute of Pathology, Paracelsus Medical University Salzburg, Salzburg, Austria; ^20^ Elisabethinen Hospital, Linz, Austria; ^21^ Department of Internal Medicine V (Haematology and Oncology) Innsbruck Medical University, Innsbruck, Austria; ^22^ 3rd Medical Department, Hanusch Hospital, Vienna, Austria; ^23^ Institute of Pathology, Ludwig-Maximilians University, Munich, Germany; ^24^ Department of Hematology, Oncology, and Clinical Immunology, Heinrich-Heine-University, Düsseldorf, Germany; ^25^ Stanford University Cancer Institute, Stanford, CA, USA; ^26^ Department of Pathology, Hematopathology Unit and James P Wilmot Cancer Institute, University of Rochester Medical Center, Rochester, New York, USA

**Keywords:** myelodysplasia, diagnostic criteria, standardization, pre-MDS conditions

## Abstract

Myelodysplastic syndromes (MDS) comprise a heterogeneous group of myeloid neoplasms characterized by peripheral cytopenia, dysplasia, and a variable clinical course with about 30% risk to transform to secondary acute myeloid leukemia (AML). In the past 15 years, diagnostic evaluations, prognostication, and treatment of MDS have improved substantially. However, with the discovery of molecular markers and advent of novel targeted therapies, new challenges have emerged in the complex field of MDS. For example, MDS-related molecular lesions may be detectable in healthy individuals and increase in prevalence with age. Other patients exhibit persistent cytopenia of unknown etiology without dysplasia. Although these conditions are potential pre-phases of MDS they may also transform into other bone marrow neoplasms. Recently identified molecular, cytogenetic, and flow-based parameters may add in the delineation and prognostication of these conditions. However, no generally accepted integrated classification and no related criteria are as yet available. In an attempt to address this challenge, an international consensus group discussed these issues in a working conference in July 2016. The outcomes of this conference are summarized in the present article which includes criteria and a proposal for the classification of pre-MDS conditions as well as updated minimal diagnostic criteria of MDS. Moreover, we propose diagnostic standards to delineate between ´normal´, pre-MDS, and MDS. These standards and criteria should facilitate diagnostic and prognostic evaluations in clinical studies as well as in clinical practice.

## INTRODUCTION

Myelodysplastic syndromes (MDS) comprise a heterogeneous group of myeloid neoplasms defined by peripheral cytopenia, bone marrow (BM) failure, morphologic dysplasia in one or more hematopoietic lineages, and genetic instability with increased risk to transform to secondary acute myeloid leukemia (AML) [1-4]. Morphologic dysplasia represents a major diagnostic criterion of MDS and can be detected in erythroid cells, neutrophilic cells and megakaryocytes. However, it is often difficult to define the degree of dysplasia and there is always a certain inter-observer variability when dysplastic cells are counted on BM smears.

A first classification of the MDS was introduced by the French-American-British (FAB) cooperative working group [5, 6]. This proposal was based on cytomorphologic criteria and served as standard for many years. Between 2001 and 2016, the World Health Organization (WHO) created updated versions of this proposal [7-9]. However, within WHO categories of MDS, the course and prognosis vary significantly among patients, depending on age, co-morbidities, karyotype, somatic mutations, and epigenetic features of the dominant clones [1-4, 10-12]. In addition, most cytogenetic and molecular markers, although being useful in the diagnosis and prognostication of MDS, are not specific for MDS, but are also found in other BM neoplasms. Moreover, many mutations identified in patients with MDS may also be detected in healthy elderly individuals [13-15]. Other patients have persistent cytopenia of unknown etiology without morphologic or cytogenetic evidence of MDS or other underlying pathologies, a condition termed idiopathic cytopenia of undetermined significance (ICUS) [16-19].

Although all these conditions are potential pre-phases of MDS, they may also develop into other hematopoietic neoplasms or may just persist without clinical manifestations [13-19]. It is therefore important to develop prognostic parameters (predicting MDS evolution) and to define these conditions using solid criteria and a generally accepted (suitable) nomenclature. Several of the recently discovered cytogenetic, molecular, and flow cytometry-based markers may help in the delineation and prognostication of these conditions. However, although preliminary proposals for the definition and classification of some pre-MDS conditions have been published [15-17] no generally accepted criteria or classification are as yet available.

In 2006, an international working group made a first attempt to address the issue of potential pre-MDS conditions [16]. In an effort to refine these definitions and to discuss the biology, terminologies, and criteria of pre-MDS conditions, these experts met again in Vienna in a working conference in 2016 (July 1-3). The outcomes of this conference are summarized here and include proposed criteria for a classification of pre-MDS conditions as well as a proposed update for minimal diagnostic criteria of MDS. In addition, new diagnostic standards are discussed. Details about the conference and the consensus discussion are described in the supplement.

## DEFINITION OF MDS AND MINIMAL DIAGNOSTIC CRITERIA

MDS are characterized by peripheral cytopenia, BM failure, morphologic dysplasia in one or more myeloid lineages, and genetic instability with increased risk to transform to AML. The term ´syndrome´ indicates clinical relevance and thus implies that cytopenia is an important diagnostic feature. In most patients, a straightforward diagnosis of MDS can be made on the basis of WHO criteria [7-9]. However, in some patients with cytopenia, it may be difficult to establish or exclude overt MDS. These are patients without cytogenetic anomalies and/or only mild cytopenia, patients with an abnormal karyotype and mild cytopenia but no overt dysplasia, or patients with transfusion-dependent macrocytic anemia without karyotypic anomalies, molecular markers, or diagnostic dysplasia. To assist in these cases, minimal diagnostic criteria have been proposed by the WHO in 2001 and 2008 [7, 8] and by our working group in 2007 [16]. Our group is of the opinion that these criteria are still valid but need slight adjustments, based on novel markers and improved diagnostic approaches. Proposed revised (updated) minimal diagnostic criteria sufficient to establish the diagnosis of ´MDS´ are presented in Table 1. MDS are characterized by BM failure with peripheral cytopenia and cyto-morphologic dysplasia in erythroid cells and/or neutrophils and/or megakaryocytes. Prerequisite criteria, which must be fulfilled, include i) persistent cytopenia for at least 4 months (unless a blast cell excess and MDS-related cytogenetic abnormalities are present: in these patients the diagnosis can be established without delay) and ii) exclusion that another underlying condition serves as a primary cause (trigger) of cytopenia and/or dysplasia. The degree of cytopenia that qualifies as a criterion of MDS has been a matter of debate [16, 20-22]. Analyses of studies including the MDS databases which generated the international prognostic scoring system (IPSS) and its revision, the IPSS-R, have demonstrated that use of standard hematologic values to define cytopenic cutoffs for MDS diagnosis may be more appropriate than the WHO-recommended thresholds, also being cognizant of conditional (age-, altitude-, sex-, and ethnic-related) blood count variations [22]. Thus, our faculty is of the opinion that standard hematologic values should be employed to define cytopenia levels needed for the diagnosis of MDS. In the absence of cytopenia (defined by local institutional ranges), however, MDS should not be diagnosed. In these patients, a pre-MDS condition may be identified.

**Table 1:** Proposed minimal diagnostic criteria of MDS*

**A. Prerequisite Criteria** (both must be fulfilled)
- Persistent (4 months) peripheral blood cytopenia** in one or more of
the following lineages: erythroid cells, neutrophils, platelets
(exception: in the presence of a blast cell excess and MDS-related cytogenetic
abnormalities the diagnosis of MDS can be established without delay)
- Exclusion of all other hematopoietic or non-hematopoietic disorders
as primary reason for cytopenia/dysplasia***
**B. MDS-Related (Major) Criteria** (at least one must be fulfilled)
- Dysplasia in at least 10% of all cells in one of the following lineages
in the bone marrow smear: erythroid; neutrophilic; megakaryocytic****
- ≥15% ring sideroblasts (iron stain)
or ≥5% ring sideroblasts (iron stain) in the presence of *SF3B1* mutation
- 5-19% myeloblasts on bone marrow smears (or 2-19% myeloblasts on blood smears)
- Typical chromosome abnormality(ies) by conventional karyotyping or FISH*****
**C. Co-Criteria** (for patients fulfilling A but not B, and otherwise show typical
clinical features, e.g. macrocytic transfusion-dependent anemia; two or more of
these co-criteria must be fulfilled for considering a provisional diagnosis of MDS)
- Abnormal findings in histologic and/or immunohistochemical studies of bone marrow
biopsy sections supporting the diagnosis of MDS****
- Abnormal immunophenotype of bone marrow cells by flow cytometry, with
multiple MDS-associated phenotypic aberrancies indicating the presence of a
monoclonal population of erythroid and/or myeloid cells
- Evidence of a clonal population of myeloid cells determined by
molecular (sequencing) studies revealing MDS-related mutations******

*The diagnosis of MDS can be established when both prerequisite criteria (´A´) and at least one major criterion (´B´) are fulfilled. If no major criterion is fulfilled, but the patient is likely to suffer from a clonal myeloid disease, co-criteria (´C´) should be applied and may help in reaching the conclusion that the patient has a myloid neoplasm resembling MDS or will develop MDS. In this diagnostic setting, repeated bone marrow investigations during follow-up may be required to arrive at a final diagnosis of MDS.

**Cytopenia defined by local institutional reference values.

***As more and more patients with two co-existing bone marrow neoplasms are diagnosed, it is important to state that in rare cases, MDS can be diagnosed even if another co-existing disease potentially causing cytopenia is also detected.

****Examples: clusters of abnormally localized immature precursors (ALIP); clusters of CD34+ blast cells; dysplastic micromegakaryocytes detected by immunohistochemistry (≥10% dysplastic megakaryocytes).

*****Typical chromosome abnormalities are those recurrently and typically found in MDS patients (e.g. 5q-, -7) and considered as indicative of MDS by the WHO even in the absence of morphologic criteria of MDS.

******Detection of multiple mutations typically seen in MDS (e.g. SF3B1) increases the likelihood that the patient suffers from MDS or will develop MDS.

Abbreviations: MDS, myelodysplastic syndrome(s); FISH, fluorescence in situ hybridization; WHO, World Health Organization; Hb, hemoglobin; ANC, absolute neutrophil count.

Major (MDS-related) criteria include i) dysplasia of at least 10% of cells in one or more major BM lineage(s) (erythroid, neutrophilic, megakaryocytic) or an increase in ring sideroblasts (RS) of ≥ 15% (or ≥ 5% in the presence of a *SF3B1* mutation), ii) an increase in myeloblasts of 5-19% in dysplastic BM smears (in the absence of AML-specific gene rearrangements) or 2-19% myeloblasts in peripheral blood smears, and iii) a MDS-related (5q-, -7, complex, etc.) karyotype (Table 1). At least one of these major MDS criteria has to be met (together with pre-requisite-criteria) to arrive at the diagnosis of MDS. When MDS-related (major) criteria are not fulfilled but the patient exhibits typical clinical features (e.g. macrocytic transfusion-dependent anemia) and no other underlying disease can be detected, the diagnosis of MDS can still be considered (can be provisionally proposed) when certain MDS co-criteria are met. These co-criteria include typical histologic and immunohistochemical findings, typical multi-parameter flow cytometry (MFC) patterns, and typical somatic mutations (Table 1). The minimal allele burden required to count as a co-criterion of MDS remains uncertain. Whereas a minimum burden of 2% was used as a working definition of clonal hematopoiesis with indeterminate potential (CHIP) [15], the allele burden is usually higher in MDS (often > 10%) (Table 1). The presence of multiple mutations (typically seen in MDS, e.g. *SF3B1*) increases the likelihood that the patient suffers from a myeloid neoplasm resembling MDS or will develop MDS during follow up. An impaired BM function, as demonstrated by a reduced number of colony-forming progenitor cells (CFU), was also proposed as co-criterion of MDS in our initial proposal [16]. However, in most centers, the CFU assay is not performed routinely. Therefore, this co-criterion, although helpful, was removed in our updated proposal. BM histology and immunohistochemistry results (e.g. megakaryocyte dysplasia or a blast cell increase) may also support the diagnosis of MDS [23-25]. Therefore, a definitive conclusion by the hematopathologist that BM histology- and/or immunostaining results are consistent with MDS are now also included as a co-criterion (Table 1). When all evaluations are negative and the patient is classified as ICUS, the recommended standard is to follow the clinical course and to perform laboratory blood parameters regularly. A repeat BM examination is recommended depending on laboratory findings and the clinical course (as in low risk MDS patients) [16].

## POTENTIAL PRE-PHASES OF MDS

During the past decade, several interface- and potential pre-MDS conditions have been proposed, including ICUS, idiopathic dysplasia of unknown significance (IDUS), clonal cytopenia of unknown significance (CCUS) and CHIP [15-19]. It is important to note that these conditions may i) persist without clinical manifestations, ii) progress to MDS after a variable time period, iii) progress to another myeloid neoplasm, or iv) progress to another hematologic or even non-hematologic disease. Therefore, the appendices ´US´ (for undetermined/unknown significance) or IP (indeterminate potential) are appropriate in these definitions. Table 2 shows a summary of these conditions, together with specific features and proposed defining criteria. In the following paragraphs, their features and clinical impact are reviewed.

**Table 2:** Pre-MDS and MDS conditions: typical features and criteria

	Pre-MDS Conditions and MDS					
Feature	ICUS	IDUS	CHIP	CCUS	LR MDS	HR MDS
Monoclonal/ Oligoclonal						
	-/+	+/-	+	+	+	+
Dysplasia*	-	+	-	-	+	+
Cytopenia(s)**	+	-	-	+	+	+
BM blasts	<5%	<5%	<5%	<5%	<5%	<20%
Flow abnormalities	+/-	+/-	+/-	+/-	++	+++
Cytogenetic abnormalities						
	-/+***	-/+***	+/-	-	+	++
Molecular aberration/s****						
	-	-	+	+	++	+++

*At least 10% of all cells in a given lineage (erythroid, neutrophil, or megakaryocyte) are dysplastic.

**Persistent cytopenia(s) recorded over a time-period of at least 4 months.

***In a subset of cases, a small-sized clone with MDS-related anomaly is detectable by FISH.

****A molecular aberration is defined by MDS-related mutations and an allele burden of ≥2%. The working definition for pre-MDS conditions is also ≥2% allele burden, whereas the minimal allele burden to count as a co-criterion of MDS should be higher (e.g. 10%). However, a high allele burden does not exclude the presence of CHIP or CCUS. It is also important to note that in most patients with MDS, multiple gene mutations/aberrations are found. When several co-criteria of MDS are present, the diagnosis MDS can be established in the absence of diagnostic dysplasia.

Abbreviations: MDS, myelodysplastic syndrome(s); ICUS, idiopathic cytopenia of undetermined significance; IDUS, idiopathic dysplasia of undetermined significance; CCUS, clonal cytopenia of undetermined significance; LR, low risk; HR, high risk; BM bone marrow, FISH, fluorescence in situ hybridization.

### Idiopathic cytopenia of undetermined (unknown) significance (ICUS)

ICUS is defined by cytopenia of any degree in one or more lineages: erythrocytes, neutrophils, or platelets. The cytopenia has to be i) persistent (≥ 4 months), ii) lacking minimal diagnostic criteria of MDS and iii) not explained by any other hematologic or non-hematologic disease (Supplementary Table S1) [16, 19]. In some ICUS patients, the type of cytopenia (e.g. transfusion-dependent macrocytic anemia) may point to the potential of MDS or a MDS-prephase. Patients with ICUS are further subdivided into ICUS-A (anemia), ICUS-N (neutropenia), ICUS-T (thrombocytopenia), and ICUS-PAN (bi/pancytopenia) (Supplementary Table S2) [19]. The precise clinical implication of this classification remains to be determined. Apart from MDS, differential diagnoses to ICUS-N include, among others, drug-induced neutropenia, chronic hepathopathies, autoimmune disorders, and cyclic neutropenia. ICUS-T has to be separated from immune-mediated thrombocytopenia (ITP) where platelets are usually dropping quickly to very low levels, whereas this is not the case in ICUS-T. In patients without a clear diagnosis but otherwise suggestive (MDS-related) features, further tests and markers should be applied to confirm or exclude BM failure and the presence of a clonal cell population (e.g. by fluorescence *in situ* hybridization [FISH], MFC, and molecular analyses). Depending on the specific clinical situation and laboratory features, specific molecular tests and markers (example: *JAK2* V617F in the case of BM fibrosis) should be applied [16, 19, 25]. The clinical course in patients with ICUS is variable. Some of these patients will progress to MDS or AML [16-19, 26]. Other patients may develop a lymphoproliferative neoplasm or mast cell neoplasm. In some patients, a small-sized clone is initially detected by FISH, MFC, or molecular analyses [18, 27-29]. These patients may have a higher risk to transform into MDS or another BM neoplasm. As soon as MDS-related molecular aberrations are detected in a patient with ICUS, the diagnosis may change to CCUS (mutation with ≥ 2% allele burden and no other signs or co-criteria of MDS present) or MDS (other MDS criteria also present) (Table 2).

### Idiopathic dysplasia of undetermined (unknown) significance (IDUS)

More and more patients are referred with no, slight, or only transient cytopenia, but peripheral blood (PB) abnormalities resembling dysplasia (band cells, Pseudo Pelger forms, hypogranulated neutrophils, unexplained macrocytosis). In several of these cases, BM examinations show mild to marked signs of dysplasia in one or more lineages [17, 19, 30]. In the absence of any detectable cytopenia and absence of any cytogenetic or molecular abnormalities, this condition should be called IDUS. In fact, a number of reactive conditions and other pathologies can provoke mild or even marked dysplasia in normal (polyclonal) BM cells, with or without cytopenia, and in several of these cases, a reactive non-hematopoietic disease or a deficiency syndrome (e.g. copper deficiency) may be identified during follow-up (Table 3). Therefore, the term IDUS seems justified. However, as soon as persistent cytopenia and other MDS-related criteria are also detected, the diagnosis changes to MDS. The definition of IDUS is shown in Supplementary Table S3.

**Table 3:** Causes of bone marrow (BM) cell dysplasia and cytopenia

Cause / Etiology	Marker / Diagnostics*
Inherited bone marrow failure syndromes	Family history and genetic tests
Myeloid neoplasms affecting the BM**	Hematologic diagnosis (WHO criteria)
Lymphoproliferative neoplasms (T-LGL)	Hematologic diagnosis (WHO criteria)
Paroxysmal nocturnal hemoglobinuria (PNH)	PI-linked antigens (*PIG-A* mutation)
Pure red cell aplasia (PRCA)	Reticulocyte count - dynamics
Gelatinous BM transformation	Case history, BM histology
(=serous atrophy of bone marrow)	
Vitamin B12 or folate deficiency	Serum vitamin measurements
Copper deficiency	Case history, serum copper measurement
Viral infections (CMV, EBV, HIV,	Virology
parvo virus B19, hepatitis, others)	
Bacterial infections	Bacteriology
Visceral leishmaniasis	Parasitology and cytology
Autoimmune disorders	Immunology diagnostic algorithms
Other chronic inflammatory processes	Inflammation-related parameters
Chronic kidney failure	Nephrology diagnostic algorithms
Chronic liver diseases***	Hepatology diagnostic algorithms
Drug-induced BM reactions	Drug intake - case history
Chemotherapy	Oncologic case history
BM stem cell transplantation	-
Radiation damage	Measurements to estimate radiation exposure
Toxic damage (various toxins)	Toxicology

*For all differential diagnoses, a detailed investigation of the BM by cytology, histology, immunohistochemistry (and where relevant flow cytometry), is required.

**Apart from MDS, most other myeloid neoplasms affecting the BM can also produce mild or even marked BM cell dysplasia. In many cases, long-term therapy with cytoreductive agents promotes the dysplasia.

***Chronic liver diseases, such as chronic hepatitis or alcohol-induced hepathopathies leading to liver cirrhosis, are typically associated with neutropenia and thrombocytopenia.

Abbreviations: BM, bone marrow; T-LGL, T cell large granular lymphocyte leukemia; CMV, cytomegaly virus; EBV, Epstein Barr virus; HIV, human immunodeficiency virus.

### Clonal hematopoiesis of indeterminate potential (CHIP)

Since cancer is generally caused by the serial acquisition of multiple mutations, the appearance of an initial somatic mutation in a gene that drives myeloid malignancies in a healthy hematopoietic stem cell may lead to clonal expansion in the absence of overt malignancy [31, 32]. In fact, hematopoietic (stem) cells can acquire somatic lesions (mutations) during the lifetime of a healthy individual, the vast majority of which confer a neutral or negative effect on the survival and expansion of the stem cell, without definitive signs of a BM neoplasm [13-15]. As a result, somatic mutations that are otherwise typically found in MDS or other BM neoplasms, are detected in a subset of healthy individuals, and the prevalence of such clones increases with age [13-15, 33]. The term CHIP was created to define the state in which a somatic genetic alteration in a gene that is recurrently mutated in hematologic malignancies is identified in an otherwise healthy individual [15]. Although initially discussed in the context of MDS, CHIP must be regarded as a more general phenomenon. In particular, individuals with CHIP have an increased risk of acquiring a hematologic malignancy, and may progress to MDS, but may also develop other myeloid or even lymphoid neoplasms. It has also been shown that CHIP is associated with an increased risk to develop therapy-related myeloid neoplasms [34-36]. However, not all patients with CHIP develop an overt malignancy during their lifetime.

With regard to the minimal clone size, a first proposal was that the mutant allele burden in the PB should be ≥ 2% to call a condition CHIP [15]. Our group is of the opinion that this proposal should qualify as a general working definition of CHIP, provided that the other criteria of CHIP are also fulfilled. The full definition of CHIP includes the absence of persistent (≥ 4 months) cytopenia and exclusion of other underlying conditions as primary reason for the observed mutation(s) in a non-cytopenic patient (Table 2, Supplementary Table S4). For example, the preliminary diagnosis of CHIP would change into systemic mastocytosis as soon as mastocytosis is detected in a BM biopsy (even if no cytopenia is present). An unresolved question is whether evidence of clonal expansion of any type of hematopoietic cells should qualify as ´indicative of CHIP´. Individuals with CHIP who have the potential to develop MDS will likely have mutations that are known to be recurrently mutated in MDS [15]. It is not yet clear whether some of these MDS-related mutations have a higher prognostic impact than others with regard to progression to MDS or overall survival [37]. Therefore, we are of the opinion that CHIP should (for the moment) not be sub-classified into prognostically different groups (or terms). Finally, the term CHIP should only be used for individuals who have a normal blood count.

### Clonal cytopenia of unknown significance (CCUS)

The term CCUS has been suggested for patients in whom cytopenia and clonal abnormalities are found, but no dysplasia is seen and no other clonal BM neoplasm is detected (Table 2) [15]. Our group is of the opinion that CCUS should only apply when all investigations have excluded MDS, i.e. no MDS-related morphologic or MFC abnormalities and no MDS-related cytogenetic lesions (like 5q-) are found. In other words, in the presence of either MDS-related criteria or definitive (multiple) co-criteria, the diagnosis CCUS may change to MDS. Likewise, in a patient with cytopenia and typical MFC pattern or typical immunohistochemical findings (e.g. megakaryocyte dysplasia), a provisional diagnosis of MDS may be established even if the clonal marker is not specific for MDS (example: *TET2* mutation with high allele burden). The term CCUS should thus be reserved for rare conditions where no or only slight ( < 10%) BM dysplasia and no definitive cytogenetic, histologic or MFC-based signs of MDS are detected (Supplementary Table S5). With regard to the allelic burden, the same working definition should apply as for CHIP ( ≥ 2%). In the presence of multiple mutations, the likelihood that the patient has or will develop MDS is higher. Another important differential diagnosis to consider is a myeloid neoplasm with germline predisposition, a new entity recently proposed by the WHO.

## THE BM AND PB SMEAR: STANDARDS AND RECOMMENDATIONS

The examination of representative and appropriately prepared and stained BM and PB smears remains an essential diagnostic approach in suspected MDS [4-8, 16, 38-42]. For proper morphologic assessment, well-prepared thin films from sufficient material and an appropriate stain (e.g. Pappenheim, Romanowsky, or Wright-Giemsa) are required [16]. At least 500 nucleated cells should be counted in the BM smear. In each case, BM cellularity, the erythroid-to-myeloid (E:M) ratio, and the percentage of blast cells should be recorded [9, 16, 39]. When at least 10% of cells in the erythroid, neutrophil, or megakaryocyte lineage(s) are dysplastic, the diagnosis of MDS can be established provided that cytopenia is present and other diseases have been excluded as reason for dysplasia and cytopenia. An iron stain (with Perls´ reagent) should be performed in all cases in order to identify and count RS. Because 5% is the lower cut-off limit to diagnose cytopenia with RS in the presence of an *SF3B1* mutation (MDS-RS) [9, 41], a minimal number of 100 nucleated red cells should be examined. In the absence of an *SF3B1* mutation, the demonstration of ≥ 15% RS is indicative of erythroid dysplasia and supports the diagnosis of MDS-RS. In both instances, the diagnosis MDS can be established in a cytopenic patient (RS+) even if morphologic dysplasia is only seen in < 10% of cells in the three major BM lineages.

Guidelines for the detection, classification, and enumeration of blast cells and RS in the BM smear in MDS have been published recently [38-40]. Megakaryocytes often display signs of morphologic dysplasia in MDS. However, even in well-prepared BM smears, the number of megakaryocytes may be too low to define a percentage of dysplastic cells. Therefore, megakaryocytic dysplasia is often defined histologically in BM biopsy sections. Monocytes may be increased in number in BM smears and may be quite immature. If monocytes are particularly immature, it may be difficult to discriminate them from blast cells by morphology. Erythroid cells and their progenitors usually show dysplasia in MDS, which should be reported in qualitative terms (mild, moderate, severe) and quantitatively as percent count. Blast cells should be counted as percent of all nucleated BM cells independent of erythroid predominance. In other words, many cases formerly diagnosed as AML FAB M6 based on erythroid predominance (where blast cells were counted as percentage of all nucleated BM cells after subtraction of erythroid cells) are now classified as MDS, based on the lower blast count in the total BM cell compartment [9, 41, 43]. It is also important to examine well-prepared and appropriately stained PB smears in all cases with suspected or known MDS and to report morphologic features (e.g. absence of granulation) and the percentages of various cell types, including Pseudo-Pelger forms, hypogranulated neutrophils, blast cells, and monocytic cells [16]. The number of reticulocytes should also be determined in all patients with suspected or known MDS.

## BONE MARROW HISTOLOGY AND IMMUNOHISTOCHEMISTRY (IHC): CURRENT STANDARDS

In all patients with suspected MDS, a thorough investigation of appropriately processed and stained BM biopsy sections by histology and IHC should be performed [16, 23-25]. In (suspected) pre-MDS conditions, histologic investigation is required to exclude an overt BM neoplasm and other disorders, such as gelatinous transformation, infection, or BM carcinosis. In patients with established MDS, BM histology and IHC provide important diagnostic information and/or may reveal prognostic features, including BM fibrosis, a focal increase in CD34+ progenitors, increased angiogenesis, a hypocellular BM or concomitant mastocytosis (Table 4) [23-25]. The evaluation and enumeration of CD34^+^ progenitor cells and CD117/KIT^+^ cells by IHC in BM biopsy sections is important and can confirm morphologic results in uncertain cases where e.g. BM smears were contaminated with PB [16, 23-25].

**Table 4:** Value and impact of BM histology and immunohistochemistry (IHC) in MDS

Indication - Differential Diagnosis	Key (IHC) markers
Diagnosis of hypocellular (hypoplastic) MDS	Cellularity, CD34
Diagnosis of MDS-U	-
Separation from AML when smears are of poor	CD34 (CD117/KIT*)
quality or blood-contaminated	
Separation from hypoplastic AML	CD34 (CD117/KIT*), cellularity
Separation from aplastic anemia	Cellularity, CD34
Separation from lymphoproliferative disorders	T cell / B cell markers
Diagnosis of a concomitant (underlying) mastocytosis	KIT, tryptase
Multifocal accumulations of progenitor cells	CD34
Abnormal distribution/localization of progenitor cells**	CD34 (CD117/KIT*)
Abnormal accumulation and morphology	CD42b, CD61, CD31
(dysplasia) of megakaryocytes***	
Demonstration of bone marrow fibrosis	Gömöri´s silver impregnation
Demonstration of increased angiogenesis	CD31, CD34

*An increase in CD34+ cells by IHC can assist in the quantification of the progenitor (blast) cell compartment in MDS. In the case that BM blast cells lack expression of CD34, CD117/KIT should be employed as an alternative stain for the visualization and enumeration of immature precursor cells.

**The previously used term ´atypical localization of immature precursor cells (ALIP)´ is obsolete and should no longer be used.

***In many patients with MDS, demonstration of megakaryocyte dysplasia is only possible by a histologic and immunohistochemical investigation of the BM. Note that immature megakaryocytes (megakaryoblasts) may only be detectable by IHC.

Abbreviations: BM, bone marrow; AML, acute myeloid leukemia; IHC, immunohistochemistry; MDS-U, unclassifiable MDS.

BM biopsy specimens are usually taken from the posterior iliac spine and should be of adequate length ( ≥ 1.5 cm) [16, 25]. The specimen should be fixed in neutral formalin (or alternative standard fixation), decalcified in EDTA (at least 8 hours), and embedded in paraffin-wax [16, 25]. Ideally 1 µm-thin sections should be performed, however, up to 3 µm is acceptable. Standard routine stains include hematoxylin-eosin (H&E), Giemsa, Prussian blue, AS-D chloroacetate esterase (CAE), and Gömöri´s silver impregnation. CAE is of value for the detection of minor alterations of the microarchitecture of the BM, including minute infiltrates of CAE-negative cells (e.g. myeloblasts) [23]. Cellularity of the BM should be reported according to published proposals [44, 45]. For routine purposes, it is recommended that the pathologist determines the cellularity as ´normocellular´, ´hypocellular´, or ´hypercellular´, based on an age-adapted estimate [46].

The application of IHC is recommended in all patients with (suspected) MDS [16, 23-25]. The minimal IHC-panel should include CD34 (stem/progenitor cells), CD117/KIT (progenitor cells and mast cells), a megakaryocyte marker (like CD42b or CD61), and tryptase (mast cells, immature basophils) (Supplementary Table S6) [16, 25]. In difficult cases, additional lineage-specific antibodies such as CD3, CD14, or CD20, should be applied, depending on the differential diagnoses (Table 3). When employing CD34 as a progenitor-related IHC marker in MDS, it is important to know that endothelial cells also express this antigen. Another important point is that, in some patients with MDS, progenitor cells may be CD34-negative. In such cases, KIT/CD117 should be applied as alternative marker (Table 4). Nevertheless, in most cases, a straightforward approach is to stain and count progenitor cells (blasts) using antibodies against CD34 [23, 25, 47, 48]. Megakaryocyte markers enable the enumeration of immature and mature megakaryocytes as well as detection of atypical accumulations (grouping, clustering) and cytomorphological dysplasia of these cells [25, 49]. For the detection of monocytic cells, CD14 is a preferred IHC marker (Table 4). In case of very immature (CAE-negative) cells, CD14 and other monocytic markers (together with CD34) may help discriminate CMML from AML. Tryptase and CD117 are useful IHC markers to detect BM mast cells which are increased in almost all patients with MDS and may show spindle-shape appearance. If spindle-shaped mast cells are prominent and/or form compact clusters in the BM and/or express CD25, it is appropriate to perform mutation analysis of *KIT* (especially *KIT* D816V). In such cases, a coexisting mastocytosis (occult mastocytosis) is often detected (Table 4) [16, 25, 50].

## KARYOTYPING IN MDS: CURRENT RECOMMENDATIONS AND STANDARDS

Conventional karyotyping of BM cells should be performed in all patients with (suspected) MDS or pre-MDS. It is required by current recommendations to examine at least 20 metaphases [51]. When a clear-cut result is obtained even 10-20 metaphases may be sufficient. Reporting of karyotypes should be performed using ISCN guidelines [52]. As per definition, a clone is defined by two or more metaphases showing the same gain or structural rearrangement (such as deletion, inversion or translocation) of chromosomal material or at least three metaphases showing loss of the same chromosome [52]. When no cell growth or no sufficient result can be obtained, FISH should be performed [16, 53, 54]. Combined application of conventional karyotyping and FISH increases the rate of detected chromosome abnormalities [54]. FISH analysis should cover at least the following regions: 5q31, cep7, 7q31, 20q, cep8, cepY and p53, being cognizant of the limitation of this method in not detecting all karyotypic abnormalities and for some probes not being MDS-defining (20q, cep8, cepY, p53). More recently it has been shown that FISH is a suitable alternative to serial BM investigations [55, 56]. In fact, FISH analysis of enriched CD34^+^ blood cells is a sensitive approach that provides relevant cytogenetic information if BM is not available and may also be used for prognostication [55, 57]. If available, multi-color FISH can be applied, for example to decipher complex abnormalities or define unclear marker-chromosomes [58]. In general, almost all karyotype abnormalities in MDS are considered to represent somatic defects. In rare cases, however, there may be suspicion of a constitutional inborn defect. In these patients, T lymphocytes or non-hematopoietic cells, such as cultured fibroblasts should be examined. In up to 30% of patients with MDS, clonal evolution and the presence of one or more subclones is reported. A subclone is defined by detection of additional chromosomal defects (apart from the primary chromosome defect) in at least 2 cells (or 3 cells for monosomies) and absence of these additional defects in the other clonal cells. A complex karyotype is defined by at least 3 chromosome defects in one cell population (one clone) [51, 52]. The recognition of clonal evolution in MDS patients over time is clinically relevant [58-60]. Therefore, karyotyping of BM or PB CD34^+^ cells should be repeated during follow-up in patients with suspected progression. Although the definitive clinical impact of acquired additional chromosomal defects in MDS remains unknown, karyotypic evolution is in general associated with disease progression and a poor prognosis [60, 61]. In addition, it is generally appreciated that cytogenetics at diagnosis remains a most important prognostic approach [62-64]. Therefore, karyotyping has been included in all relevant prognostic scoring systems. An overview of the most frequently detected cytogenetic abnormalities is shown in Supplementary Table S7.

## MUTATION PROFILES IN MDS AND PRE-MDS: CURRENT STANDARDS AND LIMITATIONS

Cancer evolution is a stepwise process characterized by the acquisition of molecular lesions and other defects, resulting in clonal evolution and subclone formation, a related molecular diversification as well as clonal expansion [32, 65, 66]. The earliest steps of cancer evolution in MDS are expected to show just one or a few somatic mutations in driver genes that are recurrently mutated in myeloid malignancies, without histological abnormalities or cytopenia. Indeed, it has been reported that several disease-related mutations in critical target genes, such as *DNMT3A* or *TET2*, are detectable in a subset of healthy individuals (CHIP) or those with ´skewed´ hematopoiesis without an overt MDS or AML [13-15]. These cases increase in prevalence with age and are defined as CHIP [15]. It can be expected that the numbers of diagnoses of CHIP will increase substantially as sequencing studies are applied broadly in daily practice. Some of these mutations may be associated with a substantial risk of transformation into MDS over time, whereas other mutations may be associated with a lower risk. However, only a few studies have addressed this issue so far [37]. The likelihood of evolution into MDS or AML in the follow-up may increase with the number of mutations detected and their allele burden.

In patients with overt MDS, numerous sequencing studies have been conducted in the recent past [67-76]. In these studies, it turned out that i) recurrent mutations are detectable in a majority of patients with MDS and ii) mutation profiling data can confirm the diagnosis of MDS [70-76]. Therefore, we are of the opinion that the documented presence of MDS-related mutations in BM or PB cells should be regarded as a new co-criterion for a provisional diagnosis of MDS. Mutation profiling in MDS is also of prognostic significance [73-76]. Because of the many recurrent mutations in MDS, next generation sequencing (NGS) strategies are generally required [70-77]. Recently, comprehensive myeloid marker panels have been established and are used in clinical practice (Table 5) [70-77]. Some of these panels may be more specific for MDS and pre-MDS conditions whereas others may cover all myeloid neoplasms, including MDS. All in all, NGS profiling can be regarded as a new standard technique to define molecular aberration profiles in patients with suspected or established MDS. In some of these patients, the mutations detected may point to the presence of an overlap syndrome or a concomitant BM neoplasm. For example, the presence of *JAK2* V617F will raise the suspicion of an MPN/MDS [78, 79], and detection of *KIT* D816V is usually associated with an underlying mastocytosis [23, 50]. Relevant mutations detected in MDS are shown in Table 5.

**Table 5:** Somatically mutated genes detectable in patients with MDS and CHIP

Gene Abbreviation	Gene Name	Chromosome Location	Frequency*	
			MDS	CHIP
*NRAS*	Neuroblastoma RAS oncogene	1p13.2	+/-	-
*DNMT3A*	DNA-methyltransferase 3 alpha	2p23	+	+
*SF3B1*	Splicing factor 3b, subunit 1	2q33.1	+	+/-
*IDH1*	Isocitrate dehydrogenase 1	2q33.3	+/-	-
*GATA2*	GATA binding protein 2	3q21.3	+/-	-
*KIT*	V-kit oncogene homolog	4q11-12	+/-	-
*TET2*	Tet methylcytosine deoxygenase 2	4q24	+	+
*NPM1*	Nucleophosmin	5q35.1	-	-
*EZH2*	Enhancer of zeste homolog 2	7q35-36	+/-	-
*JAK2*	Janus Kinase 2	9p24	+/-	+
*CBL*	CBL proto-oncogene	11q23.3	+/-	+/-
*KRAS*	Kirsten sarcoma viral oncogene	12p12-11	-	-
*ETV6*	Ets variant 6	12p13	-	-
*FLT3*	Fms-related tyrosine kinase 3	13q12	-	-
*IDH2*	Isocitrate dehydrogenase 2	15q26.1	-	-
*TP53*	Tumor protein p53	17p13.1	+/-	+/-
*PRPF8*	Pre-mRNA processing factor 8	17p13.3	-	-
*SRSF2*	Serine/arginine-rich splicing factor 2	17q25.1	+	+/-
*CEBPA*	CCAAT/enhancer-binding protein A	19q13.1	-	-
*ASXL1*	Additional sex combs like 1	20q11	+	+
*U2AF1*	U2s nuclear RNA auxiliary factor 1	21q22.3	+/-	-
*RUNX1*	Runt-related transcription factor 1	21q22.12	+/-	-
*BCOR*	BCL6 Corepressor	Xp11.4	-	-
*ZRSR2*	Zinc finger (CCCG type)	Xp22.1	+/-	-
*STAG2*	Cohesin complex factor	Xq25	+/-	-

Gene names refer to standard nomenclature. MDS, myelodysplastic syndromes; CHIP, clonal hematopoiesis of indeterminate potential.

*Score of frequency: -, <1%; +/-, 1-10%; +, >10% of all patients.

The presence of multiple mutations typically seen in MDS (e.g. *SF3B1, SRSF2*) increases the likelihood that the patient suffers from MDS or will develop MDS.

## FLOW CYTOMETRY IN MDS: STANDARDS AND LIMITATIONS

A number of previous and more recent studies have shown that MFC can assist in the diagnosis and prognostication in MDS [80-87]. In cases with suspected MDS or pre-MDS conditions MFC may help in reaching the conclusion that BM cells are abnormal and/or immature, and that the cells examined express an aberrant immunophenotype suggesting the presence of a myeloid neoplasm. In addition, MFC can be employed to quantify (CD34^+^) progenitor cells, erythroid progenitors, neutrophils, and/or monocytic precursor cells. Evaluation of immunophenotypic features of BM cells in patients with suspected MDS can yield three principal results, i.e. i) absence of MDS-related features, ii) inconclusive anomalies, and iii) anomalies highly consistent with a clonal myeloid neoplasm such as MDS [88]. In fact, although no marker-abnormality and no abnormal marker profile is MDS-specific, an accumulation of such changes may help in discriminating normal/reactive BM from clonal conditions. The likelihood of a myeloid neoplasm (MDS) increases with the number of phenotypic aberrations detected [80-85]. However, the final diagnosis of MDS has to be based on additional (clinical and laboratory) criteria. A summary of recurrent immunophenotypes detected in myeloid cells in MDS is shown in Table 6. Apart from its value as diagnostic tool, immunophenotyping can also assist in the prognostication in MDS [86-91]. In addition, MFC may improve currently available prognostic scoring systems [92-95]. An obvious disadvantage of MFC is that no generally accepted consensus concerning the optimal standard-protocol and technique to apply exists. Current efforts and ongoing multi-center projects have the aim to standardize and harmonize methodologies and reagents, in order to increase the general impact and awareness of this important approach and to facilitate its use in daily practice [96]. Flow cytometry might also be useful for predicting responses to therapy in MDS [97, 98].

**Table 6:** Recurrent immunophenotypic abnormalities detected by flow cytometry in MDS

CD34^+^ progenitor cells
- increase in CD34^+^ cells*
- absolute and relative (to all CD34^+^) decrease in number of
CD34^+^/CD10^+^ or CD34^+^/CD19^+^ cells (hematogones)
- abnormal expression of CD45, CD34 or CD117
- abnormal granularity (sideward light scatter)
- overexpression or lack of expression of CD13, CD33, or HLA-DR
- expression of ´lymphoid´ antigens: CD5, CD7, CD19, or CD56
- expression of CD11b and/or overexpression of CD15
Maturing Neutrophils
- decreased granularity (sideward light scatter)
- abnormal distribution of immature and mature subsets
- lack of or abnormal expression of CD11b, CD13 or CD33
- delayed expression of CD16 or lack of CD10
- expression of CD56
Monocytes
- lack of or abnormal expression of CD13, CD14, CD16, or CD33
- abnormal expression of CD11b or HLA-DR
- overexpression of CD56
- abnormal granularity or distribution of immature and mature subsets
Erythroid precursor cells
- decreased or heterogeneous expression of CD36 and CD71
- abnormal frequency of CD117^+^ erythroid precursors
- abnormal frequency of CD105^+^ erythroid precursors
- abnormal CD105 fluorescence intensity

*An increase in CD45^+^/CD34^+^ cells by flow cytometry can assist in the quantification of the progenitor (blast) cell compartment in MDS. In cases where blast cells lack CD34, CD117/KIT can be employed as alternative marker of progenitor (blast) cells. MDS, myelodysplastic syndromes

## IMPACT OF PRE-MDS CONDITIONS ON DAILY PRACTICE AND HEALTH CARE STRATEGIES

Sequencing studies of PB or cell-free plasmatic DNA are increasingly used to examine either germline predisposition to disease or for early diagnosis of cancer. Many such studies will identify CHIP, leading to an increasing number of referrals to hematology centers, and managing these referrals may become a challenge. In this regard, it will be important to implement the terminologies proposed herein (CHIP, CCUS) in daily practice. In addition, it is important to standardize the marker-panels and techniques applied to detect CHIP and CCUS. One important issue is the optimal germline control. Proposed germline controls are buccal swabs, hair follicles, nails, and cultured fibroblasts. Our faculty is of the opinion that reports providing information about genomic aberrations (or exome profiles) must include precise information regarding the sequencing technology and the analytic approach as well as information concerning the germline control if examined. The variant allele fraction should also be reported. However, other questions also remain. Should all affected individuals undergo a BM examination? Should CHIP and CCUS ´carriers´ be informed that they are in a potential pre-phase of an overt blood cell disorder? Should management recommendations include avoidance of potential mutagenic events, such as smoking or radiation, in these cases? These questions remain open and can only be addressed appropriately in forthcoming observational studies. Finally, an important question is whether and how health care systems will be able to pay for the investigations, referrals, follow-up evaluation and management.

## SCORING SYSTEMS IN MDS: UPDATE AND RECOMMENDED STANDARDS

Although more and more prognostic variables have been identified in the context of MDS and the updated WHO classification [9, 99] estimation of the clinical course and survival remains a clinical challenge. In order to address this challenge, a number of different scoring systems have been developed in the past. Until 2012, the IPSS served as a golden standard of prognostication [100-102]. However, in 2012, a revised IPSS, called IPSS-R has been published [103]. This new score improves prognostication in individual patients with MDS. The clinical value of the IPSS-R has been confirmed in numerous studies. Therefore, our faculty is of the opinion that the IPSS-R should be regarded as new golden standard of prognostication of MDS. However, even the IPSS-R has several limitations. For example, it remains unknown whether the IPSS-R is useful in patients receiving interventional therapy or targeted drugs. In addition, there are other independent risk factors that need to be considered, such as red blood cell transfusion dependence or genetic and somatic aberrations [71-76, 101]. Especially gene aberration profiles and MFC data may add in the predictive power of prognostic scoring systems. Therefore, molecular (genetic and somatic) aberrations and MFC-based aberration profiles should be validated and integrated in forthcoming refinements of the IPSS-R.

## CONCLUDING REMARKS AND FUTURE PERSPECTIVES

The increasing number of diagnostic and prognostic parameters and assays and the advent of new therapeutic approaches in MDS are major challenges in daily practice. Moreover, more and more patients are referred in whom a potential pre-phase of MDS is diagnosed but definitive criteria of MDS are not fulfilled. Based on these developments, it is important to revisit and refine current diagnostic criteria and standards in MDS and to establish definitions and criteria for pre-MDS conditions. In the current article, we propose such definitions and criteria. In the emerging era of genome-medicine these criteria and the related terminologies may be of crucial importance and should assist in the evaluation of MDS and pre-MDS in daily practice.

## SUPPLEMENTARY MATERIALS TABLES



## Abbreviations

AML: acute myeloid leukemia
BM: Bone marrow
CAE: Chloroacetate esterase
CCUS: Clonal cytopenia of unknown significance
CHIP: Clonal hematopoiesis of indeterminate potential
FAB: French-American-British
FISH: Fluorescence *in situ* hybridization
ICUS: Idiopathic cytopenia of unknown significance
IDUS: Idiopathic dysplasia of unknown significance
IHC: Immunohistochemistry
IPSS: International prognostic scoring system
IPSS-R: Revised IPSS
MFC: multi-parameter flow cytometry
MDS: Myelodysplastic syndrome(s)
PB: Peripheral blood
RS: Ring sideroblasts
WHO: World Health Organization
