# Supplementary material for: Proposed minimal diagnostic criteria for myelodysplastic syndromes (MDS) and potential pre-MDS conditions
Source: Oncotarget. 2017 Jul 5;8(43):73483–500. doi: 10.18632/oncotarget.19008 (PMC5650276; doi:10.18632/oncotarget.19008)
Supplement: Supplementary file 1 [file oncotarget-08-73483-s001.pdf]

Proposed minimal diagnostic criteria for myelodysplastic syndromes (MDS) and potential pre-MDS conditions

Supplementray Material

Information on the Working Conference and the Consensus Discussion

The Working Conference on MDS and Pre-MDS conditions (official title: Standards and Standardization in Myelodysplastic Syndromes) was organized in Vienna in July 2016 (July 1-3, 2016). The related project with in-depth discussion lasted from January 2016 until December 2016. The discussion phase was split into a pre-conference phase (via e-mails and smaller preparative meetings), the conference discussion (Working Conference: 3 days), and a post-conference discussion phase (July-December 2016). The consensus discussion and the consensus decision-making process were organized in accordance with recently published guidelines.[1]

In the final discussion round, the paper-draft was discussed and adjusted based on input provided by all faculty members and available information. Open discussion points were discussed in the faculty (consensus group = co-authors) until a clear-cut result (100% of faculty members agreed) was obtained or no consensus was reached. Only those statements, criteria, and definitions that are based on a 100% consensus among all faculty members were included in the final document.

The final document and its content were approved by all faculty members (all co-authors) before submission. All actively contributing (only those) faculty members are included as co-authors on the final document.

1. Graham R, Mancher M, Wolman DM, Greenfield S, Steinberg E, eds; Institute of Medicine; Board on Health Care Services; Committee on Standards for Developing Trustworthy Clinical Practice Guidelines. Clinical Practice Guidelines We Can Trust. Washington, DC: National Academies Press; 2011.

Supplementary Tables

Supplementary Table S1

Revised Criteria for Idiopathic Cytopenia of Undetermined Significance (ICUS)

- Persistent cytopenia (≥4 months) in one or more peripheral blood cell lineages

- Minimal diagnostic criteria of MDS not fulfilled (see Table 2 in main document)

- All other causes of cytopenia also excluded*

- No molecular aberration (mutation) detected in myeloid cells**

- No or only minor flow cytometric aberrations in immature and mature erythroid and myeloid cells***

---

*Investigations needed to establish the diagnosis ICUS include:
- Detailed case history (inherited cytopenia, infection, toxins, drugs, mutagenic events)
- Thorough clinical investigations including x ray and sonography of spleen
- Differential blood count (microscopic) and complete serum chemistry
- Bone marrow histology and immunohistochemistry
- Bone marrow smear including an iron stain
- Flow cytometry of bone marrow and peripheral blood cells
- Conventional chromosome analysis and FISH (5q31, cep7, 7q31, cep8, p53, others)
- Next generation sequencing (NGS) studies
- Exclusion of viral infections (HCV, HIV, CMV, EBV, others)
- Follow up investigations (depending on the course)

**As soon as clonal aberrations (otherwise found in hematopoietic neoplasms) are detected (allele burden ≥2%) the diagnosis changes from ICUS to CCUS.

***performed according to ELN guidelines.

Supplementary Table S2

Proposed Classification of ICUS

Proposed Term	Suggested Abbreviation	Definition (ICUS criteria fulfilled*)
Idiopathic Anemia of Uncertain Significance	ICUS-A	Persistent anemia<br>PLT normal<br>ANC normal
Idiopathic Neutropenia of Uncertain Significance	ICUS-N	Hb normal<br>PLT normal<br>Persistent neutropenia
Idiopathic Thrombocyto-penia of Uncertain Significance	ICUS-T	Hb normal<br>Persistent thrombocytopenia<br>ANC normal
Idiopathic Bi/Pancytopenia of Uncertain Significance	ICUS-PAN**	Persistent anemia and/or<br>Persistent thrombocytopenia and/or<br>Persistent neutropenia

*ICUS criteria: the patient has persistent cytopenia for at least 4 months, MDS criteria are not met, and no other reason/underlying disease that could explain persistent cytopenia has been found. **two or 3 cytopenias are required to establish ICUS-PAN.
Abbreviations: ICUS, idiopathic cytopenia of undetermined significance; ICUS-A, ICUS with anemia; PLT, platelet count; ANC, absolute neutrophil count; ICUS-N, ICUS with neutropenia; Hb, hemoglobin; ICUS-T, ICUS with thrombocytopenia; ICUS-PAN, ICUS with bi/pan-cytopenia.

Supplementary Table S3

Revised Criteria for Idiopathic Dysplasia of Undetermined Significance (IDUS)

- No persistent peripheral blood cytopenia (≥4 months) demonstrable*

- Persistent dysplasia (≥4 months) in at least 10% of cells in one or more of the major bone marrow lineages (erythroid, neutrophilic, megakaryocyte)

- Minimal diagnostic criteria of MDS not fulfilled (see Table 2)

- All other causes of bone marrow dysplasia also excluded (see Table 4)

- No molecular aberration (mutation) detected in myeloid cells**

- No or only minor flow cytometric aberrations in immature and mature erythroid and myeloid cells***

---

*As soon as persistent cytopenia develops the diagnosis changes from IDUS to MDS.
**As soon as MDS-related molecular aberrations are found (allele burden ≥2%) but cytopenia is still not detectable, the diagnosis changes from IDUS to CHIP.
***according to ELN guidelines.

Supplementary Table S4

Proposed Criteria for Clonal Hematopoiesis of Indeterminate Potential (CHIP)

- One or more molecular aberrations (mutations otherwise found in patients with MDS or other myeloid neoplasms) detected in bone marrow or peripheral blood cells*

- No persistent peripheral blood cytopenia (≥4 months) demonstrable**

- Minimal diagnostic criteria of MDS not fulfilled (see Supplementary Table S5)

- Other underlying hematopoietic and non-hematopoietic diseases also excluded

---

*The working definition of CHIP includes an allele burden of ≥2%; mutations typically detected in CHIP, CCUS, and MDS are shown in Table 5.
**As soon as persistent cytopenia develops the diagnosis changes from CHIP to either CCUS (allele burden ≥2% and no other MDS-related criteria found) or MDS (allele burden ≥10% and/or other MDS-related features found).

Supplementary Table S5

Proposed Criteria for Clonal Cytopenia of Undetermined Significance (CCUS)

- One or more molecular aberrations (mutations otherwise found in patients with MDS or other myeloid neoplasms) detected in bone marrow or peripheral blood cells*

- Persistent cytopenia (≥4 months) in one or more peripheral blood cell lineages

- Minimal diagnostic criteria of MDS not fulfilled (see Table 2 in main document)**

- All other causes of cytopenia and molecular aberration also excluded***

---

*The working definition of CHIP includes an allele burden of ≥2%; mutations typically detected in CHIP, CCUS, and MDS are shown in Table 5.
**Neither any of the MDS-related criteria nor any of the MDS-co-criteria are fulfilled (except MDS-related mutation/s) – otherwise the diagnosis changes to MDS.
***Recommended investigations are listed in the legend to Supplementary Table S1.

Supplementary Table S6

Recommended Immunohistochemical Markers in MDS

A: Minimal Panel

marker(s)	cell type(s)
- CD34*	blast cells, progenitors, endothelial cells
- CD31	megakaryocytes, endothelial cells
- CD42b, CD61	megakaryocytes
- KIT (CD117)*	blast cells, progenitor cells, mast cells

B: Extended Panel – according to the cell lineage to be examined

marker(s)	cell type(s)
- CD3	T cells
- CD71, E-Cadherin	erythroid cells
- CD14**	monocytes
- CD15	neutrophils and monocytes
- CD20	B cells
- CD25	B cell subset, atypical mast cells
- CD38, CD138	plasma cells
- CD68, CD68R**	monocytes, macrophages, myeloid cells
- 2D7, BB1	basophils
- Tryptase*	mast cells, immature basophils

*In a very few cases of MDS, blasts cell may be CD34-negative cells. In these patients, CD117 can be applied as an alternative, whereas tryptase usually is negative or shows only a weak reactivity with blast cells.

** Monocyte/macrophage markers may be helpful to discriminate between immature monocytic cells and myeloblasts (CMML versus AML).

Supplementary Table S7

Overview of most frequently detected chromosome abnormalities in MDS

Karyotype - abnormality	Frequency in MDS (% of positive cases)*	Diagnostic value (MDS criterion)
5q−	15%	++**
−7/7q−	11%	+
+8	8-9%	+
−18/18q−	3-4%	+/-***
20q−	3-4%	+/-
−5	3%	+***
−Y	3%	-
+21	2%	+/-
−17/17p−	2%	+/-***
inv/t(3q)	2%	+
−13/13q−	2%	+/-
+1/+1q	2%	+/-
−21	1-2%	+/-
+11	1-2%	+/-
−12	1-2%	+/-***
12p−	1%	+/-
11q−	1%	+/-
9q−	1%	+/-
−20	1%	+/-***

*Data were obtained from the available literature - see:
Haase et al, Ann Hematol 2008;87: 515-26.
**An isolated 5q- is indicative of a distinct variant of MDS according to WHO criteria.
***Close association with complex abnormalities.
MDS, myelodysplastic syndrome.
